# Severe inflammatory reaction induced by peritoneal trauma is the key driving mechanism of postoperative adhesion formation

**DOI:** 10.1186/1471-2482-11-30

**Published:** 2011-11-14

**Authors:** Sergei V Pismensky, Zhomart R Kalzhanov, Marina Yu Eliseeva, Ioannis P Kosmas, Ospan A Mynbaev

**Affiliations:** 1 Laboratory of Pathophysiology, Faculty of Basic Medicine and Laboratory of Fermentative Fibrinolysis, Faculty of Biology, M.V. Lomonosov Moscow State University, Lomonosovsky Prospekt 31-5, Moscow, 117192, Russia; 2 The Experimental Research & Modeling Division, Moscow State University of Medicine & Dentistry, Delegatskaya str 20/1, Moscow, 127473, Russia; 3 Department of Obstetrics and Gynecology, the Institute of Advanced Training of the Federal Medical-Biological Agency of Russia, Volokalamsky road 30, Moscow, 123182, Russia; 4 The Institute of Reproductive Technologies AltraVita, Nagornaya 4A, Moscow, 117186, Russia; 5 Department of Pediatrics, Faculty of Pediatrics, A.D. Asfendiyarov Kazakh National Medical University, Tole bi street 88, Almaty, 050012, The Republic of Kazakhstan; 6 Laboratory of Physiology, Faculty of Medicine, University of Ioannina, Ioannina, 45110, Greece

## Abstract

**Background:**

Many factors have been put forward as a driving mechanism of surgery-triggered adhesion formation (AF). In this study, we underline the key role of specific surgical trauma related with open surgery (OS) and laparoscopic (LS) conditions in postoperative AF and we aimed to study peritoneal tissue inflammatory reaction (TIR), remodelling specific complications of open surgery (OS) versus LS and subsequently evaluating AF induced by these conditions.

**Methods:**

A prospective randomized study was done in 80 anaesthetised female Wistar rats divided equally into 2 groups. Specific traumatic OS conditions were induced by midline incision line (MIL) extension and tissue drying and specific LS conditions were remodelled by intraperitoneal CO_2 _insufflation at the 10 cm of water. TIR was evaluated at the 24^th^, 72^nd^, 120^th ^and 168^th ^hour by scoring scale. Statistical analysis was performed by the non-parametric t test and two-way ANOVA using Bonferroni post-tests.

**Results:**

More pronounced residual TIR was registered after OS than after LS. There were no significant TIR interactions though highly significant differences were observed between the OS and LS groups (p < 0.0001) with regard to surgical and time factors. The TIR change differences between the OS and LS groups were pronounced with postoperative time p < 0.05 at the 24^th ^and 72^nd^; p < 0.01 - 120^th ^and p < 0.001 - 168^th ^hrs. Adhesion free wounds were observed in 20.0 and 31.0% of cases after creation of OS and LS conditions respectively; with no significant differences between these values (p > 0.05). However larger adhesion size (41.67 ± 33.63) was observed after OS in comparison with LS (20.31 ± 16.38). The upper-lower 95% confidential limits ranged from 60.29 to 23.04 and from 29.04 to 11.59 respectively after OS and LS groups with significant differences (p = 0.03). Analogous changes were observed in adhesion severity values. Subsequently, severe TIR parameters were followed by larger sizes of severe postoperative adhesions in the OS group than those observed in the LS group.

**Conclusions:**

MIL extension and tissue drying seem to be the key factors in the pathogenesis of adhesion formation, triggering severe inflammatory reactions of the peritoneal tissue surrounding the MIL resulting in local and systemic consequences. CO_2 _insufflation however, led to moderate inflammation and less adhesion formation.

## Background

Adhesions are an important health care problem [1-5], causing long term postsurgical complications such as infertility, pelvic pain and bowel obstructions, Therefore, a broad spectrum of approaches has been tested to prevent postsurgical adhesion formation albeit with unequivocal results [6,7].

Laparoscopy (LS) has been established as the golden standard for the surgical treatment of a variety of benign tumors and other pathologic conditions. Laparotomy or open surgery (OS) is increasingly being regarded as outdated and thus may not be the treatment of choice of many pathologic conditions in the abdominal and pelvic cavities for much longer. Many studies have been comparatively evaluating perioperative changes, as well as short and long term outcomes of OS versus LS [8-10]. In order to describe and calculate the mobility of the abdominal wall and the nature of the underlying disturbances, Stumpf et al [11] used three-dimensional stereography, which is a noninvasive optical method of measuring surface areas. They measured pre- and post-surgery abdominal wall mobility in patients undergoing LS and OS surgery and found a significant difference in abdominal wall mobility between patients treated by LS in comparison with those treated by OS. Abdominal movement was completely recovered the 7^th ^day after LS, whereas a significant lack of mobility was still observed the 12^th ^day after OS. Consequently, the minimal invasive approach presented a positive effect on abdominal wall integrity.

It is well known that LS, compared with OS, reduces adhesion formation. Therefore, many contradicting findings have been presented and discussed concerning LS-related postoperative adhesion formation mechanisms [12]. Generally accepted mechanisms of adhesion formation after OS, including tissue ischemia and decreased tissue plasminogen activator (tPA activity) with subsequent transition of persistent fibrinoid adhesions (deposits) to permanent fibrous adhesions were automatically copied to LS [13-16]. CO_2 _insufflation was presumed a co factor of adhesion formation since during laparoscopic procedures surgeons can perform manipulations due to the creation of CO_2_-pneumoperitoneum [17,18].

However, both OS and LS have their specific traumatic effects on the abdominal wall and peritoneum tissue. OS has more additional traumatic effects related with the midline incision line (MIL) giving access to the operated organs, tissue drying, direct hand-manipulations, accumulation of foreign bodies and severe tissue ischemia by MIL extension, ligations and suturing of the abdominal wound. Most of these tissue traumatic factors are reduced or excluded during LS with subsequent beneficial outcome such as fast postsurgical recovery, less morbidity, pain decrease etc. [9,10,19]. Therefore, the starting point of our study was that we should inflict the same initial abdominal wall trauma to two groups of rats. Then we would perform OS in one group and LS in another. We presumed that the more pronounced impact of OS on postoperative complications, such as adhesion formation, would be clearly defined in the models of the OS with MIL extension and tissue drying and aimed to study peritoneal tissue inflammatory reaction (TIR), remodelling specific complications of OS versus LS and subsequently evaluating of adhesions induced by these conditions.

## Methods

### Animals

The experimental protocol was approved by the M.V. Lomonosov Moscow State University Review Board and Animal Care Committee as a part of the research project of MD thesis by SVP.

The animals were kept under standard laboratory conditions at a temperature between 20 and 25°C, and a relative humidity of 40 to 70%. They had a day cycle of 14 h light and 10 h dark, a standard laboratory diet and free access to food and water. The animals were housed at the Laboratory for Animal Care, Faculty of Basic Medicine, M.V. Lomonosov Moscow State University, Moscow, Russia).

### Experimental design

The experiment was done by a blind randomization in anaesthetised spontaneously breathing 80 adult female 6 month old Wistar rats weighing between 210-230 g divided equally into 2 groups (Table 1): laparotomy or OS group and LS group. 19 rats were excluded from final analysis since they died before the first evaluation (10 from OS and 9 - LS). Animals were euthanized at the 24^th^, 72^nd^, 120^th ^and 168^th ^hours after surgery with intramuscular injection of toxic doses (100 mg/kg) of Thiopental Sodium and the severity of the peritoneal inflammatory reactions was studied by a scoring system. Postsurgical adhesions were evaluated in all animals examined after 168 hours of surgery.

**Table 1:** Design of surgical procedures and evaluation methods in both OS and LS groups

Adhesion inducing trauma	Simulation of basic specific surgical conditions during 1 hr	Time of evaluation and type of results	
		
		24-72-120-168 hrs	168 hrs
MIL	MIL extension	Postsurgical follow up of inflammation severity	Postsurgical adhesions' frequency and size
			
	CO_2 _insufflation at 10 cm of water		

### Anaesthesia and surgical procedures

Anaesthesia was achieved and maintained by intramuscular fractional injection of thiopental sodium in the musculus femoralis (50 mg/kg) and inhalation of air.

According to our study design, surgical procedures included similar adhesion inducing trauma i.e. MIL, in the first step in both groups. Then MIL extension was performed in the OS group as a simulation of basic specific traumatic conditions related with laparotomy. CO_2 _was insufflated in the LS group as a main specific condition is related with laparoscopy.

In the OS group a 2 cm MIL was performed by scalpel and the abdominal cavity was kept open for 1 hour with extension of the MIL by eye retractor. The same MIL was performed in the LS group to induce adhesion formation in the trocar sites and 18 g catheter "HELMFLON^®^/HELMSYTE^®^" of the company HELM India PVT Ltd was fixed in the middle of the MIL. Consequently, this MIL was closed without extension. CO_2 _was insufflated during 1 hour through this catheter to simulate laparoscopic conditions during CO_2 _pneumoperitoneum and the wound around this catheter was considered as a port-site wound. The MIL was closed by two layers of continuous sutures of vicril 5/0 (Ethicon, Johnson & Johnson) in both groups. The first suture layer included peritoneum, musculus and fascia; the second layer - only the skin.

### CO_2_-pneumoperitoneum setup

**Figure 1:**
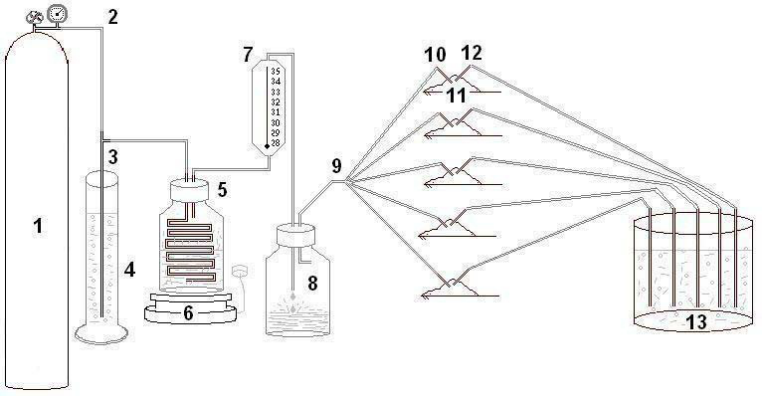
**Setup of system to creation of CO_2_-pneumoperitoneum (Definitions in the text)**.

To insufflate CO_2 _a special setup (Figure 1) was designed, consisting of the CO_2 _balloon (1), a T-figurative metal tube with different tubes connected to different devices including, connection tubes (2, 3) a water valve (4). This setup also includes the humidifier (5) and heating device (6) with thermometer (7) and the excess water reservoir (8).

The heating device, the thermometer and the excess water reservoir controlled the temperature and humidity of the CO_2 _gas. The temperature in this system was kept at 37°C. The temperature in animals' body was kept by the permanent flow of warmed and humidified CO_2 _to keep intraperitoneal pressure level at the 10 cm of water. Animals' body temperature was above 35°c outside of their skin. Excess water after condensation accumulated in the special reservoir, and warm and humidified CO_2 _gas was administered through a distributor (9) with 5 small tubes connected with the let in 18 G catheter (10) which was inserted into the rats' abdomen (11).

Insufflation pressure was controlled and monitored with two water valves. The first water valve (4), which was situated next to the CO_2 _balloon and controlled the pressure in the insufflation system. The abdominal cavity of the animals was connected through the outlet 20 G catheter (12) with a second water valve (13) to monitor the intra-abdominal pressure. When the intraperitoneal pressure was achieved 10 cm of water excess of CO_2 _was deflated by means of the second water valve (13). The CO_2_-pneumoperitoneum was simultaneously created simultaneously in 5 animals.

### Evaluation of macroscopic changes and adhesion formation

The severity of the peritoneal inflammatory reactions was studied by a scoring system (Table 2) in 5 rats in each group at the first three time points 24, 72 and 120 hr and in 16 and 15 animals in OS and LS groups respectively at the 168^th ^hr after surgery. A sum of individual inflammation parameter scores was calculated as the total inflammation score for each animal and its mean and standard deviation (SD) values in OS and LS groups were analyzed in different postsurgical time points.

**Table 2:** Macroscopic residual inflammatory reaction scoring system.

Parameters		Scores
Smooth wound surface with palish or palish-pink color of serosa		0.5
	
Hyperemia	Dilated vessels	0.5
	
	Petechial extravasation/hemorrhage	0.5
	
	Hematoma	0.5
	
	Hemorrhagic imbibition	0.5

Black blue color of wound		0.5

Edema		0.5

Fibrinoid deposits on the wound surfaces		0.5

Necrotic tissue and detritus		0.5

Fester and other changes		0.5

Total score		

To objectively present the relief of adhesions in the abdominal and pelvic cavity we carefully evaluated the frequency and character of adhesions on the laparotomy line, on the uterine horns, on the area of the peritoneal adhesion formation model and on the other abdominal and pelvic structures/organs. These data were recorded by a researcher blinded to the treatment groups. The adhesion size was observed as follows: 0 - no adhesions; 1-25%; 26-50%; 51-75% of traumatized area or total (76-100%) involvement. Adhesion severity was recorded as follows: 0 - no adhesions; 1 - no resistance to separation; 2 - little resistance to separation; 3 - moderate resistance (force required) to separation; 4 - sharp dissection needed to separation.

### Randomization

Groups were formed randomly and after the creation of a model, the assistants marked the animals. After filling in the individual protocol of surgical procedures for each animal, these protocols were each put in a separate envelope, which was sealed immediately. After 168 hr, the animals were randomly picked for the evaluation. One outsider surgeon (MYuE) and OAM managed this procedure. Each animal was separately evaluated simultaneously by two researchers. There were only 5 cases of disarrangements and those cases were repetitively evaluated to find consensus. Then, a new protocol of adhesion formation for each animal was filled in and, finally, after the experiment had ended, it was matched with the first protocol which was filled after the previous surgery.

### Statistics

Statistical analysis was performed by Graph Pad Prism. Mean ± SD is indicated unless stated otherwise. P values were obtained by two-way ANOVA using Bonferroni post-tests for repeated inflammation values and the unpaired t test for adhesion frequency, size and severity. We performed the Kolmogorov & Smirnov normality test to find out data sampled from populations that follow Gaussian distribution. Data concerning adhesion size as well as adhesion severity passed this test, but data concerning adhesion frequency did not. Using two-way ANOVA with Bonferroni post-tests we tried to answer the following questions:

1). Does the surgery have the same effect at all values of time (24, 72, 120 and 168 hr)?

2). Does the surgery affect the result or are the curves different?

3). Does time affect the result or are the curves horizontal?

## Results

**Figure 2:**
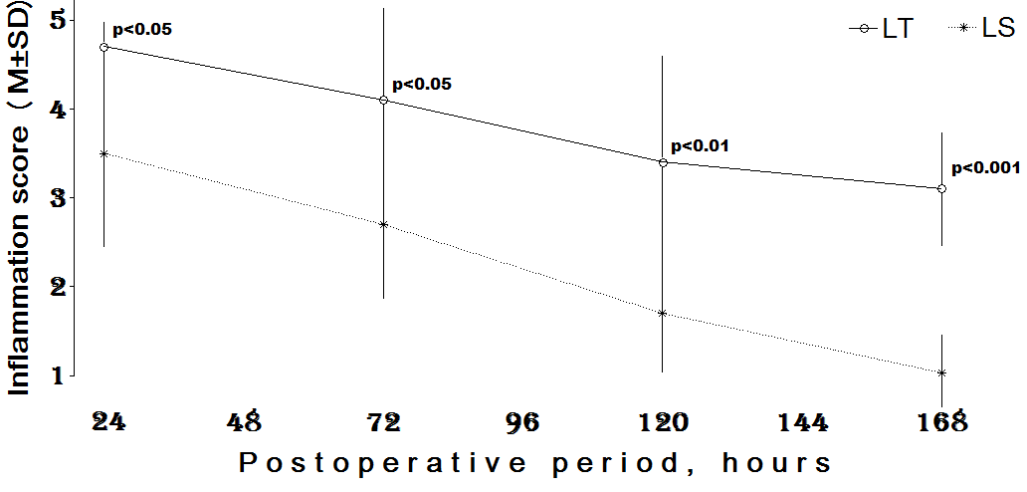
**Dynamics of postoperative inflammation score changes after induction conditions of open and laparoscopic surgery**. P value (LT vs LS) was obtained by two-way ANOVA with Bonferroni post-tests.

More pronounced residual peritoneal tissue inflammatory reaction parameters were registered after OS in comparison with those after LS (Figure 2).

1) Interaction accounts for approximately 1.61% of the total variance (F = 1.19, DFn = 3, DFd = 53 and p = 0.32). If there is no interaction overall, there is a 32% chance of randomly observing effect. Subsequently the interaction is considered not significant.

2) Surgery accounts for approximately 26.53% of the total variance (F = 58.53, DFn = 1, DFd = 53 and p = 0.0001). If the surgery has no effect overall, there is a less than 0.01% chance of randomly observing effect. Subsequently, the surgical impact is considered extremely significant.

3) Time accounts for approximately 32.33% of the total variance (F = 23.77, DFn = 3, DFd = 53 and p = 0.0001). If time has no effect overall, there is a less than 0.01% chance of randomly observing effect. The effect of time is also considered to be extremely significant.

There were no significant interactions in peritoneal tissue inflammatory reaction parameters, but highly significant differences were observed between the OS and LS groups (p < 0.0001) with regard to both surgical and time factors. The differences of changes in peritoneal tissue inflammatory reaction parameters between OS and LS groups were pronounced with postoperative time p < 0.05 at the 24^th ^and 72^nd^; p < 0.01 - 120^th ^and p < 0.001 - 168^th ^hrs.

**Figure 3:**
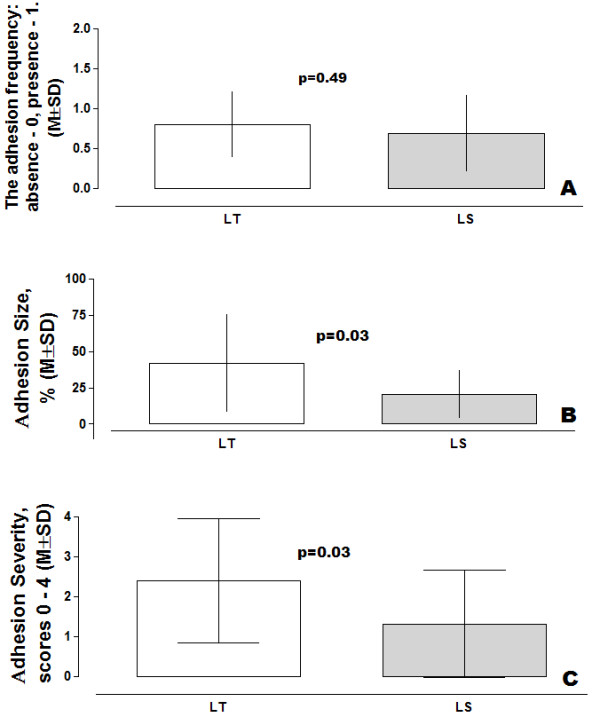
**Adhesion parameters after open (OS) and laparoscopic (LS) surgery conditions induced in the abdominal wall tissue**. (Overall unpaired t test, two-tail p value frequency, size and severity of adhesions).

The adhesion frequency was studied as follows, the presence of adhesions was considered as 1, absence - as 0 (Figure 3A). Subsequently, we found an average value of wounds covered by adhesions in 0.80 ± 0.41 and 0.69 ± 0.48 of cases after the creation of OS and LS conditions respectively. There were no significant differences between these values by unpaired t test (the two-tailed p = 0.49). However, larger adhesion size (41.67 ± 33.63%) was observed after OS (Figure 3B) in comparison with LS (20.31 ± 16.38%). The upper-lower 95% confidential limits ranged from 60.29 to 23.04 and from 29.04 to 11.59 respectively after OS and LS groups, with significant differences by unpaired t test (the two-tailed p = 0.03).

The mean value of adhesion severity (Figure 3C) was significantly higher in the OS group in comparison with the analogous parameter of the LS group: respectively 2.4 ± 1.55 and 1.31 ± 1.35 scores, with the upper-lower 95% confidential limits ranging from 3.26 to 1.54 and from 2.03 to 0.59 (the two-tailed p = 0.03). Subsequently, results for the TIR parameters ran parallel with this finding showing larger size and severe postoperative adhesions in the OS group compared with those observed in the LS group.

## Discussion

Both open and laparoscopy surgery trigger specific traumatic effects related with removing tumors or with surgical treatment of other diseases of the abdominal cavity (Table 3). On the basis of literature it is suggested that open surgery results in more additional traumatic effects due to the following conditions and complications [16,20-23]:

**Table 3:** Open and laparoscopy surgery-related factors and consequences of the surgical treatment of diseases in the abdominal and pelvic cavities

Factors	Laparotomy	Laparoscopy
**Procedures**	Midline incision Extension of the midline incision Direct hand-manipulations	Trocar or port sites CO_2 _pneumoperitoneum Indirect hand-manipulations

**Intrasurgical** **damaging factors**	Tissue drying Severe trauma Severe ischemia Accumulation of foreign bodies The possibility of bacterial contamination	CO_2 _pneumoperitoneum-related local and systemic effects: blood gas, acid base balance parameters changes, blood circulatory in large vessels and parenchymatous organs in the abdominal and pelvic cavities

**Consequences**	Painful slow recovery High morbidity Long hospitalization A big scar	Less painful fast recovery Low morbidity Short hospitalization Small scars

✓ extension of the laparotomy incision of the abdominal wall to get access to the operated organ;

✓ tissue drying due to open abdomen and prolonged surgery;

✓ direct hand-manipulations, handling of the abdominal organs and tissue;

✓ accumulation of foreign bodies - small pieces of surgical materials, tampons, plugs, napkins, suture materials

✓ severe tissue ischemia related with ligation and suturing as well as extension of the laparotomy incision of the abdominal wall;

✓ the possibility of bacterial contamination, which cannot be excluded.

However, laparoscopic surgery entails other, specific effects due to the use of gas media to extend the abdomen. From this, a large body of literature has sprung studying the pathophysiologic mechanisms of CO_2_-pneumoperitoneum induced systemic alterations such as respiratory, cardiovascular and blood gas, acid base parameters changes, as well as local disturbances in the peritoneal cavity such as decreased peritoneal pH and blood circulatory deteriorations with mesothelial hypoxemia during laparoscopic surgery [24-29]. The discussion has polarised: some claim these changes have a crucial impact on postsurgical complications such as adhesion formation and port-site cancer metastasis [30-34] others say these changes have no or little impact on postsurgical complications [16,17,28,29].

Recently, these two approaches have been systematically compared in malignant conditions in several meta-analyses. The mean operative time for LS was significantly longer but the postoperative hospital stay was shorter in comparison with those undergoing laparotomy in a meta-analysis of 2940 patients with splenectomy drawn from a large amount of publications [19]. Subsequently, it was concluded that laparoscopy is associated with a significant reduction in splenectomy-related morbidity, primarily as a function of fewer complications (pulmonary, wound, and infectious). In another meta-analysis of randomized controlled trials of LS versus laparotomy in patients with endometrial cancer, LS was associated with fewer postoperative complications, lower transfusion incidence, less blood loss, longer operation time, and shorter hospital stay [9]. Moreover, no significant differences in terms of recurrence and survival were found. Subsequently, LS was thought to be a better choice than OS if it is performed by suitably specialized surgeons in selected patients. Recently, quite striking contrasting findings were presented by Leroy et al [8] with reports of increased conversion rates and a laparoscopic colectomy risk in obese patients (BMI > 30 kg/m^2^). It was concluded that LS for left colon resections is as feasible and at least as safe in non-obese patients and the benefits of the laparoscopic approach depending on the implementation of a highly standardized surgical technique.

Most of these tissue traumatic factors are reduced or excluded during laparoscopy with the subsequent beneficial outcome. Due to the fast recovery after surgery, less morbidity, decreased pain etc, laparoscopy is now also being applied in the treatment of malignant but curable conditions resulting in equally beneficial results in the short-time follow-up for patients with malignant cancers.

More pronounced residual peritoneal tissue inflammatory reaction parameters were registered after open surgery, as compared with LS. There were no significant interactions in peritoneal tissue inflammatory reaction parameters, but highly significant differences were observed between the open surgery and LS groups (p < 0.0001) with regard to both treatment and time factors by two-way ANOVA with source of variation and Bonferroni post-tests. The differences in peritoneal tissue inflammatory reaction changes between the open surgery and LS groups were pronounced with postoperative time p < 0.05 at the 24^th ^and 72^nd^; p < 0.01 - 120^th ^and p < 0.001 - 168^th ^hrs.

We found adhesion free wounds in approximately 20.0 and 31.0% cases after creation of open surgery and LS conditions respectively. There were no significant differences between these values by unpaired t test (p > 0.05). However, larger adhesion size (41.67 ± 33.63) was observed after open surgery in comparison with LS (20.31 ± 16.38). The upper-lower 95% confidential limits ranged from 60.29 to 23.04 and from 29.04 to 11.59 respectively after open surgery and LS with significant differences by unpaired two-tail t test (p = 0.03). Subsequently, a severe peritoneal tissue inflammatory reaction arose, due to the larger size of the postoperative adhesions in the open surgery group than those observed in the LS group respectively: 2.4 ± 1.55 and 1.31 ± 1.35 scores with the upper-lower 95% confidential limits from 3.26 to 1.54 and from 2.03 to 0.59 (the two-tailed p = 0.03).

**Figure 4:**
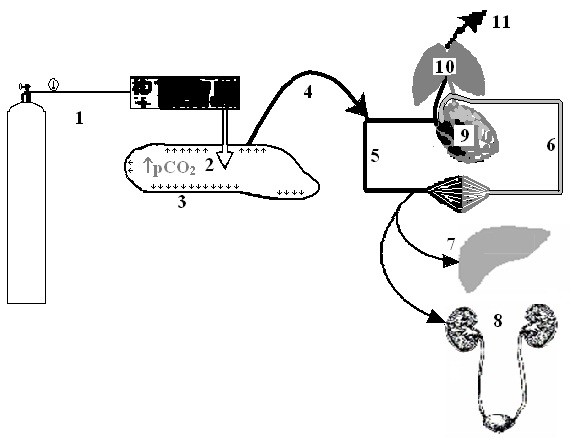
**The pathways of CO_2 _insufflation, diffusion, circulation; and elimination during laparoscopy with CO_2 _pneumoperitoneum and its pathological effects**. 1 - CO_2 _insufflation set up (including CO_2 _balloon and CO_2 _insufflator); 2 - increased tension of free CO_2 _gas (**↑pCO_2_**) in the abdominal cavity; 3 - CO_2 _diffusion into parietal peritoneum tissue (**^↑↑↑^**); 4 - passes into capillaries and vessels; 5,6 - CO_2 _accumulation and circulation in venous (5) and arterial (6) blood; 7 and 8 - circulatory and functional disturbances in parenchimateous organs (liver and kidney); 9 and 10 - cardiovascular and respiratory changes (heart and lungs); 11 - CO_2 _elimination by physiologic way.

Since excess CO_2 _is immediately eliminated through the lungs (Figure 4) by increased breathing, in our opinion CO_2 _is the most convenient physiological gas. Our results did not support the impact of CO_2_-pneumoperitoneum as a co-factor in postsurgical adhesion formation. We found severe peritoneal tissue inflammatory reaction due to surgical trauma resulting from the significantly larger size of postoperative adhesions in the open surgery group. Surprisingly, these observations are in accordance with results published by our colleagues from KULeuven [35].

## Conclusion

Midline laparotomy extension and tissue drying seem to be the key factors in the pathogenesis of postsurgical complications. They trigger severe inflammatory reactions of the peritoneal tissue surrounding the laparotomic incision resulting in local and systemic consequences, whereas CO_2 _insufflation results in moderate inflammation and less adhesion formation.

## List of abbreviations used

AF: adhesion formation; LS: laparoscopy and laparoscopic conditions; MIL: midline incision line; OS: open surgery; TIR: tissue inflammatory reaction; tPA: tissue plasminogen activator; hr: hour; hrs: hours.

## Competing interests

The authors declare that they have no competing interests.

## Authors' contributions

SVP & ZhRK - equally participated in all steps of surgical procedures, preparation of rats for anaesthesia, surgery and postsurgical animal care, as well as randomization of animals and filling in surgical protocols and interpretation of obtained results. MYuE - was involved in the surgical procedures as an outsider surgeon to blindly evaluate of inflammation and adhesions' parameters by scoring scales, and later she was also participated in the drafting of the manuscript and revising it critically for content. IPK - was involved in the design of the study, performed the statistical analysis, interpretation of results. He drafted the manuscript and revised it critically for content. OAM - as a coordinator and a principal researcher has made substantive intellectual contributions to the concept and design of this study, acquisition of data - a creation of surgical models and a blind evaluation of inflammation and adhesions' parameters by scoring scales, statistical analysis and interpretation of this study. All authors read and approved the final manuscript.

## Pre-publication history

The pre-publication history for this paper can be accessed here:

http://www.biomedcentral.com/1471-2482/11/30/prepub
